# Genetic and ecological characterization of the giant reed (*Arundo donax*) in Central Mexico

**DOI:** 10.1371/journal.pone.0319214

**Published:** 2025-05-07

**Authors:** Ricardo Colin, Erika Aguirre-Planter, Luis E. Eguiarte

**Affiliations:** 1 Departamento de Ecología Evolutiva, Laboratorio de Evolución Molecular y Experimental, Instituto de Ecología, Universidad Nacional Autónoma de México. Ciudad de México, México; 2 Facultad de Ciencias, Universidad Nacional Autónoma de México. Licenciatura de Biología. Ciudad de México, México.; Washington University, UNITED STATES OF AMERICA

## Abstract

*Arundo donax* (giant reed) is currently found in all tropical-subtropical and warm-temperate areas of the world. In Mexico, *A. donax* is a common introduced species, growing in a variety of climates and habitats. We used Inter Simple Sequence Repeats (ISSRs) markers to analyze 20 populations across different geographic regions of Mexico to estimate the geographic structure of its genetic variation, the levels of clonal diversity, and their predominant reproductive mode (clonal vs. sexual), and to explore environmental factors that may be related to genetic differentiation. In addition, we used bioclimatic variables to perform multivariate statistical analyses. We detected a total of 77 different genotypes, finding that all the analyzed populations are multiclonal (including from 3 to 9 different genotypes). The data suggest that sporadic sexual reproduction takes place in some populations. We found four main genetic groups and low levels of gene flow among clusters. Ecological characterization analyses indicate that the distribution and abundance of genotypes is structured and influenced by environmental factors, supporting the existence of three main ecological-genetic groups in Mexico (Central Highlands, Coasts, and North).

## Introduction

Understanding the factors that lead to the successful establishment of species outside their native range has been an important goal in evolutionary ecology [1]. Advances in molecular methods and statistical analyses provide great opportunities to study the patterns of ecological and evolutionary processes within and among populations in their introduced range [2]. When a species is introduced into a new range, the genetic variation within the newly founded populations is usually depleted relative to the source populations [3–8]. This spatial distribution of genetic variation is in part a product of environmental factors, including human activities, life history traits, introduction history, mating systems, and demographic history [9–11]. Studying the genetic diversity and structure of introduced populations has proven useful for understanding the evolutionary processes involved in the success of their establishment and spread into a novel range [12–15].

*Arundo* L. (Poaceae, tribe Arundineae) is a cosmopolitan genus, that according to different authors, includes three to five taxa distributed from tropical Asia to the Mediterranean Basin [16–18]. *Arundo donax,* the giant reed, has been cultivated for a long time in Asia, southern Europe, North Africa and the Middle East [16,19,20]. The native range of *A. donax* is a matter of speculation, because its biogeographic origin has been obscured through ancient and widespread cultivation. *Arundo donax* has been reported as native to southern Asia [16,21], Eastern Asia [22], or areas surrounding the Mediterranean Sea, where it occurs along other *Arundo* species, such as *A. plinii* Turra, *A. collina* Tenore and *A. mediterranea* Danin [19].

Mariani et al. [23] provided evidence that *A. donax* likely originated in Asia, and subsequently spread into the Mediterranean region. Hardion et al. [24], obtained similar results, they analyzed the hypothesis of the ancestral introduction of *A. donax* from Asia to the Mediterranean. Their results indicate that *A. donax* was represented by a single haplotype from the Mediterranean to the Middle East. The study supports a Middle East origin for the Mediterranean Basin clone of *A. donax* and was probably introduced in antiquity (before 1500 AD). In a more recent study, Zecca et al. [25] analyzed samples from a broad geographic scale; their results indicate that there are six lineages distributed from the Asian regions to the Mediterranean basin and suggest that the western and southern edges of the Qinghai-Tibet Plateau is the putative area of origin and source of ancient and cryptic lineages. The ancient spread of *A. donax* has been explained because of its multiple uses, including the basic material for making baskets, fences, fishing rods, mats, plant stakes, roof thatching, walking-sticks, musical instruments --such as the reeds for clarinets and saxophones--, shading, or as ornamental, and more recently, for erosion control and the manufacture of paper, pulp and rayon (viscose) [19,20]. For instance, in Italy *A. donax* was cultivated between the 1930s and the 1960s to obtain cellulose to produce rayon and paper [19]. Recently, this species has been identified as an energy crop, most notably as a source of bioethanol [19,26–28]. Therefore, *A. donax* became widely dispersed by humans and it is currently found growing in all the tropical-subtropical and warm-temperate areas of the world [21,23,26,29–31].

In United States, giant reed is believed to have been initially introduced into southern California from the Mediterranean in the early 1800s for erosion control, with subsequent introductions being made to Texas and Florida as late as the 1940s [16,19]. It was also used for roof thatching and cultivated to produce reeds for musical instruments [16,19]. Since its introduction to California, *A. donax* escaped cultivation and became a major invasive weed of riparian habitats, where it not only displaces native species, but in southern California also dramatically modified ecological and successional processes [16,21]. A similar process was described in Florida and along the Rio Grande on the border between Texas and Mexico [16,21,32]. Despite the invasiveness of *A. donax*, the species is propagated and sold horticulturally throughout the United States and has been planted along ditches for erosion control [30].

Although *A. donax* produces abundant flowers, viable seed has not been observed in most areas where it has been introduced [19], and asexual reproduction is presumed to be the primary mode of spread of this species by means of fragments of stems and rhizomes [21,33].

Molecular markers are an effective tool in providing informative data on the levels of genetic variation [29–31], however, the differences in the values in a given geographic area may be due to different sampling schemes and the use of different genetic markers (Table 1). For example, given the apparent lack of sexual reproduction, low levels of genetic variation within populations are expected, as has been reported for populations in the United States, South Africa, Australia and Europe, including the Mediterranean region [23,28,30,34,36,38]. On the other hand, in a study of 97 accessions collected in eight populations along the Santa Ana River in southern California, low and moderate levels of genetic diversity were reported [29]. In 203 Old World and 159 New World plants, Tarin et al. [31] found numerous genotypes and evidence for multiple introductions to the United States, with one lineage responsible for the invasion of the Rio Grande Basin, northern Mexico, and to other parts of southwest United States [31]. Genetic diversity was low in the New World and high in the Old World samples [31]. A similar value to the Old World estimate was found in Australia [37], showing that the genetic diversity is higher than that reported in the United States and Italy (Table 1). In addition, samples collected from North America and South Asia (Nepal), showed evidence of two subgroups representing North American (naturalized and cultivated) and South Asian collections [35], the result also indicated a low level of genetic diversity among the accessions, both within and between the *A. donax* subgroups (Table 1). Despite the above, molecular markers also provide critical information about the importance of the invasiveness of *A. donax*, as well as on the potential application of biological control [29–31].

**Table 1:** Comparison of the proportion of distinguishable genotypes among the Mexican populations and other regions of *Arundo donax*. *G* = Number of detected genotypes, *N *= Sample size, *G/N* = Proportion of distinguishable genotypes.

Country	G	N	G/N	Genetic markers	References
South Africa	1	40	0.025	microsatellites	[34]
Australia	1	218	0.005	AFLP	[28]
United States/Nepal	21	26	0.808	ISSR	[35]
Italy	8	86	0.093	microsatellites	[36]
New World^1^	6	159	0.038	microsatellites	[31]
Old World^2^	129	203	0.635	microsatellites	[31]
Australia	38	58	0.655	ISSRs	[37]
Mediterranean Basin	1	16	0.062	AFLP	[38]
Italy	1	12	0.083	ISSRs	[23]
Southern France	1	20	0.050	TE-based	[30]
Southern France	1	20	0.050	SRAP	[30]
United States	3	185	0.016	TE-based	[30]
United States	2	185	0.011	SRAP	[30]
United States	40	87	0.460	RAPD	[29]
United States	8	87	0.092	Isozyme	[29]
Mexico	77	449	0.171	ISSRs	This study

^1^Countries sampled and number of analyzed plants in New World: Argentina = 5, Mexico = 29, Texas/ Rio Grande Basin = 105, Southeast U.S = 12, California/Nevada = 8, [31].

^2^Countries sampled in Old World and number of analyzed plants: Spain = 132, France = 6, Portugal = 11, Italy = 24, Greece = 6, Turkey = 6, Israel = 3, Morocco = 3, Algeria = 12 [31].

In Mexico, *A. donax* is a common introduced species that is widely used for the manufacture of crafts, such as flutes, baskets, chiquihuites, floor mats, thatched roofs or as an ornamental plant (R. Colin, personal observation). It grows in a variety of climates and habitats, including disturbed marshes, wetlands, rivers, lakes, riparian zones, and along roads (R. Colin, personal observation). Despite the invasiveness of *A. donax*, there has not been a characterization of genotypes, genetic diversity or predominant reproductive mode in this country. We used Inter Simple Sequence Repeats (ISSRs), a versatile and reliable PCR-based DNA method which combines the benefits of AFLPs and microsatellites, as well as the universality of RAPDs [39–42], to study the geographic structure of genetic variation across different geographic regions in Mexico. ISSRs are genomic regions lying within the microsatellite repeats, its high reproducibility is mainly due to the nature and length of the primers (16–25 bp), allowing the use of high annealing temperatures (45–60 ºC), which ensure that only those DNA segments that are completely complementary to the primer are amplified, so, they have been successfully used to estimate genetic variation and population genetic structure of different plants, in particular clonal species [43–45]. In addition, we used multivariate statistical analyses to explore environmental factors that may be related to genetic differentiation among populations.

This study is the first report on the evolutionary ecology of *A. donax* in Mexico. Our main goals were (1) to determine the geographic pattern of genetic diversity; (2) to evaluate whether spread occurs mainly through clonal propagation or through sexual reproduction; (3) to provide information on the history of dispersal of this species; and (4) to identify genotypes and the environmental factors that determine their distribution. Our hypothesis is that, given the apparent lack of sexual reproduction in the introduced ranges of *Arundo donax*, low levels of genetic variation within populations are expected, as well as reduced genetic differentiation. Or, on the contrary, that the Mexican populations are less clonal, with more genotypes, high genetic differentiation, low levels of gene flow, and more variable than many other populations.

## Materials and methods

### Plant material

**Fig 1:**
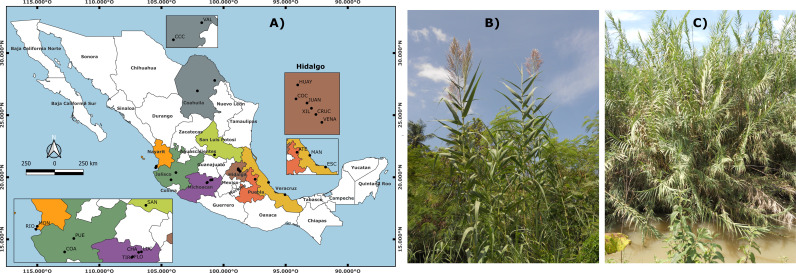
Arundo donax. In (A) geographic distribution of the populations analyzed. In (B and C) pictures of *Arundo donax* showing inflorescences, stems, and stands respectively. (B, individuals from the state of Veracruz and C, stand from the state of Jalisco, Mexico. Photographs by Ricardo Colin).

Individuals of *Arundo donax* (Fig 1) from 20 natural populations in Mexico were sampled between 2010 and 2011. We collected leaf tissues from healthy adult plants. The distance between collected plants was approximately 10 meters to reduce the probability of sampling clones or close relatives. Fresh leaf material was collected from each individual and placed in a bag for drying, followed by storage at – 80° C in the laboratory until DNA isolation.

### DNA extraction

Total DNA was extracted by grinding approximately 0.25 g of fresh leaf tissue in liquid nitrogen, and adding 600 μl of CTAB 2X extraction buffer [46], following a centrifugation at 10,000 rpm for 8 min at 4°C. The supernatant was eliminated and the sample was re-suspended in 600 μl of CTAB 2X buffer; 10 μl of RNAase (7000 u/ml) was used to digest RNA, afterwards the solution was incubated for 15 min at 37°C and then the samples were placed on ice for 15 min. After this, 40 µl of proteinase *K* (20 mg/ml) was added and incubated at 65°C for 30 min. The samples were again placed on ice for 15 min. DNA was isolated with 600 μl of chloroform: octanol 24:1. After being homogenized, the mixture was centrifuged at 12,000 rpm for 10 min at 4°C and the supernatant was transferred to a new centrifuge tube. DNA was precipitated with a 2/3 volume of ice-cold isopropanol (-20°C) and maintained for 3 h at -20°C. Then, samples were centrifuged at 13,000 rpm for 5 min and the supernatant was eliminated. The DNA was washed by inversion with 1ml of 70% ice-cold ethanol (-20°C) and recovered as a pellet by centrifugation at 8,000 rpm for 5 min at 4°C. The pellet was air-dried at room temperature and re-suspended in 45 *μl* of ultra pure water (Molecular Biology Reagent; SIGMA) and then stored at 4° C. The quality of DNA was determined by 1% agarose gel electrophoresis and concentration was quantified using an Eppendorf biophotometer.

### ISSRs amplification

A total of twenty different primers were screened from primer set # 9 from the University of British Columbia (Vancouver, Canada). Genotyping was performed with four ISSRs primers: 827(5´-ACA CAC ACA CAC ACA CG-3´); 841(5´-GAGAGAGAGAGA GAG AYC-3´; Y = C or T); 846(5´-CAC ACA CAC ACA CAC ART-3´; R = A or G) and 850 ((GT)8YC), that were selected because they produced clear and varied banding patterns in a set of samples from different localities of *A. donax*.

PCR amplifications were carried out in GeneAmp ® PCR system 2700 (Applied Biosystems, Waltham, Massachusetts, USA) in total reaction volumes of 30 µl, containing 40 ng of total DNA; 1 × PCR buffer (100mM Tris-HCL, 500 mM KCl, 10µg/ml gelatin, 1% Triton, 1.5 mg/ml BSA), 2.5 mM MgCl_2_ (for primers 841 and 846), 3 mM for primer 827, and 3.5 mM for primer 850; 0.2 mM each dNTP; 0.4 µ M of each primer, and 1 unit of *Taq* DNA polymerase. The cycling conditions consisted of an initial denaturation at 94° C for 4 min, 35 cycles of 94° C for 35 s, 52° C (primer 850), 53° C (primers 827/841), and 55°C (primer 846) for 45 s, and 72° C for 2 min followed by a final extension at 72° C for 5 min. Each PCR reaction was carried out at least twice to ensure consistency and reproducibility. A negative control was included in each amplification run, in order to control for contamination.

Amplified products were size-separated by standard horizontal electrophoresis in 2% agarose gels, using 1X TAE buffer at pH 8.0 (Tris–Acetic acid–EDTA) with a constant voltage of 160 V for 3 h at room temperature. The bands were visualized by staining with ethidium bromide and photographed under ultraviolet light using the program Kodak 1D Image Analysis Software version 3.6 (Scientific Image Systems, Eastman Kodak Company). Only the bands that showed consistent amplification were considered and molecular weights were estimated with a 100-bp DNA ladder (Invitrogen).

### Data analysis

Amplified fragments were scored in a matrix of presence (1) or absence (0) of homologous bands in each individual and for every primer. Then, a matrix was assembled by combining all loci to generate an ISSR profile (genotype) for each individual in the sample. Subsequently, we removed individuals in which no amplification was obtained in one or more primers. That is, we analyzed a total of 449 individuals of *Arundo donax*. That matrix was then used to calculate several standard measures of genotypic diversity.

### Clonal diversity

The proportion of distinguishable genotypes was measured as G/N, where G is the number of genotypes detected in a population and N is the total number of plants (ramets) analyzed [47]. We also estimated four additional measures of clonal diversity: 1) The effective number of genotypes; this measure is equivalent to the “effective number of alleles” [48]. 2) Nei’s [49] genetic diversity (expected heterozygosity) corrected for sample size. This index is also known as Simpson’s diversity index [48,50], representing the probability that two randomly chosen individuals are genetically different in the population and thus ranges from zero in a population composed of a single clone to one in a population where each sampled individual has a unique genotype. 3) The evenness index, which is an indicator of how evenly the genotypes are divided over the population [48]. This value varies from 0, where one genotype dominates and the others are represented by a single individual, to 1, in which case all genotypes in the population have uniform frequencies for each genotype, and 4) The Shannon-Wiener index corrected for sample size [51]. For the sample size correction, the number of genotypes that are only sampled once (singletons) was used to estimate the number of unsampled types [48,50]. All these analyses were carried out with the program GenoDive [50]. The percentage of polymorphic loci was estimated using the criterion of 95% with the program TFPGA version 1.3 [52].

### Genotype assignment

We used the program GenoType [50] for assigning genotypic identity to individuals. A genotype accumulation curve was assessed to determine the minimum number of loci necessary to discriminate between unique genotypes. The curve is constructed by randomly sampling loci without replacement and counting the number of observed genotypes [53]. The analysis was repeated 10,000 times for 1 locus up to *n-1* loci, creating *n-1* distributions of observed genotypes and plotting the number of genotypes detected against the number of loci, as implemented in the R package poppr version 2.2.1 [53–55].

### Comparing sexual and asexual reproduction

We estimated linkage disequilibrium in order to explore if populations are clonal (where significant disequilibrium is expected due to linkage among loci) or sexual (where linkage among loci is not expected). The null hypothesis of random mating was tested using the modified index of association rBarD [56], which is equivalent to the index of association *I*_*A*_ [57–59], but independent of the sample size [56]. rBarD is expected to be zero if populations freely recombine and significantly greater than zero if the association between alleles is non-random (clonality). To assess if loci are linked, we calculated statistics and tested significance using a permutation approach (1,000 randomization) using the poppr R package version 2.2.1 [53–55].

### Genetic structure considering all individuals

To evaluate the genetic structure among the analyzed individuals, we carried out several approaches. First, we conducted an exploratory analysis to assess the relationship between the plant individuals by means of a principal coordinate analysis (PCo) based on the Euclidean distances using the R package vegan version 2.4–4 [55,60].

Second, the Euclidean distances were used to perform an Agglomerative Hierarchical clustering analysis using the Unweighted Pair Group Method (UPGMA), as implemented in R Package stats version 3.4.1 [55]. In order to visualize the result of the hierarchical clustering analysis and to identify the clustering structure, we drew a dendrogram. To assess if the distances in the cluster tree reflect the original distances accurately, we computed the correlation between the cophenetic distances and the Euclidean distances by using the R base function cophenetic [55]. If the clustering is valid, the linking of the samples in the cluster tree should have a strong correlation with the distances between samples in the original distance matrix; values above *r* = 0.75 are considered to be a good fit. The optimal number of clusters was determined by means of the average silhouette method, which measures the quality of a clustering, determining how well each sample lays within its cluster [61]. A high average silhouette width indicates a good clustering and the optimal number of clusters *K* is the one that maximizes the average silhouette over a range of possible values for *K* [61], and the goodness of the clustering was evaluated using the silhouette width index. The silhouette analysis estimates the average distance between clusters and measures how well a sample is clustered. Samples with silhouette width (*Si*) values near one are very well clustered, small *Si* values (around 0) mean that the samples lie between two clusters, and samples with a negative *Si* are probably placed in the wrong cluster. Silhouette analyzes were performed in R software version 3.4.1 [55]. Additionally, we also performed a principal component analysis to visualize the results in a scatter plot using R-package version 3.4.1 [55].

Third, we carried out an analysis of molecular variance (AMOVA) using Arlequin version 3.5 [62] to determine how genetic variation was partitioned among clusters (groups of populations were defined according to the hierarchical clustering analysis). The significance level of the tests was obtained with 10,000 permutations.

Finally, we estimated Weir and Cockerham’s [63] coancestry coefficient (*θ*), which is analogue to Wright’s *F*_*ST*_ [64], with the software TFPGA version 1.3 [52]. To evaluate the significance level of the test, we used a jackknife over loci to obtain variance estimates, a bootstrap with 10,000 iterations over loci and confidence level of 95%.

### Genetic relationship among populations

Euclidean distances between pairs of populations were estimated with the program GenAlEx version 6.503 [65], then we used these distances to perform a subsequent cluster analysis using the Unweighted Pair Group Method (UPGMA) in R Package stats version 3.4.1 [55]. A cophenetic matrix was computed from the clustering matrix in order to assess the goodness between the matrix of Euclidean distance and the dendrogram (UPGMA method) cluster analysis.

### Ecological and genotypic characterizations

To evaluate the role of environmental conditions in populations and to detect climate differences among them, we obtained climatic data from the WorldClim database version 1.4 (http://www.worldclim.org) [66]. This database provides information about 19 bioclimatic variables generated by interpolation of climate data (average values from 1950 to 2000 period) from weather stations around the World. Bioclimatic variables are derived from the monthly temperature and rainfall values and represent annual trends (e.g., mean annual temperature, annual precipitation), seasonality (e.g., annual range in temperature and precipitation), as well as extreme environmental conditions (e.g., temperature of the coldest and warmest month) and limiting environmental factors (precipitation of the wet and dry quarters). We also included the Topography raster grid represented by elevation data from the same database records (WorldClim) [66]. We used these 20 global climate data sets for current conditions with a spatial resolution of 30 arc-seconds (~ 1 km^2^). We extracted climatic data from each georeferenced locality using DIVA-GIS version 7.5.0 [67]. We used the software R [55] to perform a principal component analysis (PCA) for an assessment of the relative positions of populations in climate space.

The genotype distribution data was related to the 20 environmental variables using canonical correspondence analyses (CCA) as implemented in the R package vegan version 2.4–4 [55,60]. The contribution of each environmental variable to the CCA model was evaluated by Monte Carlo permutation tests with forward selection at the 0.05% significance level with 10,000 permutations. The variables selected were then included in a final model for which significance was tested with 10,000 permutations.

The selected bioclimatic variables from CCA were used to estimate the Euclidean distance between populations and construct a matrix of environmental distances, which was employed to perform an Agglomerative Hierarchical clustering with the UPGMA method, as implemented in R Package stats version 3.4.1 [55]. Additionally, an ordination analysis (PCA) was carried out to visualize the results in a scatter plot using R-package version 3.4.1 [55].

## Results

### ISSR Amplification and clonal diversity

The four ISSR primers produced a total of 70 readable and reproducible bands (“loci”), ranging in size from 150 to 1500 base pairs (bp). Twenty-one loci were amplified with primer 827, eighteen with primer 841, seventeen and fourteen loci were amplified for primers 846 and 850 respectively. Of all the observed loci, 26 (25.714%) were polymorphic in the 449 analyzed individuals.

A detailed description of the values of clonal diversity for the 20 populations analyzed is given in Table 2. The proportion of distinguishable genotypes (G/N) varied among populations, with an average value of G/N = 0.280 for all populations. Xilotla, in the central state of Hidalgo, showed the lowest value (0.136), while the highest value (0.450) was found in population Santa Maria del Rio, from the state of San Luis Potosi, also central.

**Table 2:** Estimates of clonal diversity in the analyzed populations of *Arundo donax*. *N *= Sample size, *G* = Number of detected genotypes, *G/N* = Proportion of distinguishable genotypes, *EFF* = Effective number of genotypes, *DIV* = Genotypic diversity (expected heterozygosis), *EVE* = Eveness, *SHC* = Corrected Shannon-Wiener, *%P* = Percentage of polymorphic loci using the criterion of 95%, *rbarD* = Standardized Index of Association, and *p.rD* = P-values from permutation tests of *rbarD.*

Population	Code	State	N	G	G/N	EFF	DIV	EVE	SHC	%P	rbarD	p.rD
1. Huayateno	HUAY	Hidalgo	20	6	0.300	2.50	0.631	0.416	0.681	5.714	0.477	0.00009
2. Venados	VENA	Hidalgo	19	5	0.263	3.504	0.754	0.700	0.659	7.142	0.225	0.00009
3. San Juan	JUAN	Hidalgo	18	8	0.444	5.586	0.869	0.698	0.951	8.571	0.122	0.0001
4. Cococingo	COC	Hidalgo	24	4	0.166	2.071	0.539	0.517	0.455	5.714	0.094	0.098
5. Tres Cruces	CRUC	Hidalgo	22	4	0.181	3.226	0.722	0.806	0.561	2.857	0.168	0.078
6. Xilotla	XIL	Hidalgo	22	3	0.136	2.396	0.610	0.798	0.424	2.857	0.128	0.073
7. Santa Maria del Rio	SAN	San Luis Potosi	20	9	0.450	6.451	0.889	0.716	1.009	8.571	0.051	0.033
8. Atempan	ATE	Puebla	18	7	0.388	3.767	0.777	0.538	0.840	10	0.160	0.0002
9. Tiripetio	TIR	Michoacán	19	5	0.263	3.252	0.730	0.650	0.650	2.857	-0.036	0.820
10. Charo	CHA	Michoacán	19	8	0.421	5.730	0.871	0.716	0.937	8.571	0.069	0.033
11. San Lucas Pio	LUC	Michoacán	20	5	0.250	3.125	0.715	0.625	0.638	5.714	0.124	0.007
12. Florida	FLO	Michoacán	24	6	0.250	3.272	0.724	0.545	0.694	5.714	0.177	0.00009
13. Puente	PUE	Jalisco	30	9	0.300	6.428	0.873	0.714	0.931	5.714	0.014	0.276
14. Cocula	COA	Jalisco	23	9	0.391	5.813	0.865	0.645	0.961	5.714	0.117	0.001
15. Valle Cruya	VAL	Coahuila	28	8	0.285	6.426	0.875	0.803	0.895	5.714	-0.040	0.938
16.Cuatrocienegas	CCC	Coahuila	32	5	0.156	2.976	0.685	0.595	0.586	4.285	0.151	0.0002
17. Escondida	ESC	Veracruz	22	6	0.272	3.781	0.770	0.630	0.728	4.285	0.023	0.337
18. Mancha	MAN	Veracruz	23	6	0.260	3.327	0.731	0.554	0.702	2.857	-0.041	0.783
19. Rio	RIO	Nayarit	25	7	0.280	3.834	0.770	0.547	0.777	5.714	0.008	0.392
20. Montaña	MON	Nayarit	21	3	0.142	1.892	0.495	0.630	0.377	1.428	-0.084	1.00

Genetic diversity (expected heterozygosity) was high in all populations with an average value of 0.745, ranging from 0.495 in the population Montaña, located in the Pacific Coastal state of Nayarit, to 0.889 in population Santa Maria del Rio. The corrected Shannon–Wiener index, with an average of 0.723 for all populations, also supported that the Montaña population had the lowest diversity (0.377), while the Santa Maria del Rio population was the most diverse (1.009). The evenness index was high in eight populations (0.698–0.806), indicating that these populations have uniform genotype frequencies and the average value for all populations was of 0.642. The percentage of polymorphic loci varied among populations, with values ranging from 1.428% in population Montaña to 10% in Atempan population, from the central state of Puebla (Table 2).

### Genotype assignment

A total of 77 different genotypes (i.e., clones) were identified among all samples analyzed (S1 Fig). The genotype accumulation curve based on a re-sampling procedure indicated that, with 26 loci, we still did not reach the plateau of the number of genotypes expected to be present in the samples analyzed (S2 Fig), but we achieved approximately 96% of the resolution, suggesting a good approximation for determining the minimum number of loci necessary to discriminate among individuals in a population (S2 Fig).

The distribution of genotypes varied among populations and regions (S1 Fig). We found that 46 genotypes (Gen_1 - Gen_46) are distributed in the Central Highlands of Mexico, including the states of San Luis Potosi and Puebla (S1 Fig, S1 Table), 18 genotypes (Gen_47 - Gen_57; Gen_71 - Gen_77) in the Gulf Coast (state of Veracruz) and the Pacific Coast (Jalisco and Nayarit states), and 13 genotypes (Gen_58 - Gen_70) with a distribution towards North region (S1 Fig, S1 Table). From these genotypes, fifty-five unique genotypes were found among all the studied populations (S1 Table).

### Sexual and asexual reproduction

The Standardized Index of Association (rBarD) showed evidence of linkage disequilibrium among loci for 10 populations (Table 2). The observed value in these populations was significantly greater than zero (*P* < 0.05). Thus, the hypothesis of no linkage among markers was rejected for these samples, supporting a clonal mode of reproduction (Table 2). In contrast, no evidence of linkage disequilibrium among loci was found in populations Cococingo, Tres Cruces, Xilotla, Tiripetio, Valle Cruya, Escondida, Mancha, Puente, Rio and Montaña, as none of these rBarD values were significantly different from zero (P > 0.05), being consistent with (at least partially) sexual recombination. Therefore, the hypothesis of sexual reproduction for these populations was not rejected (Table 2).

To ensure that the pattern of linkage and no linkage among markers seen in the analysis was not due to a particular pair of loci, we calculated rBarD over all pairs of loci. We found both, that several loci are linked (S3 Fig), as well as evidence suggesting that there are pairs of loci that fall inside the distribution expected under no linkage (S3 Fig), indicating that these loci were freely recombining, i.e., there were virtually no associations between loci (S3 Fig).

### Genetic structure and relationships

Principal coordinate analysis (PCo) of the genetic data showed that the first two axes explained 68.72% of the total variation and suggest a general grouping according to the geographical distribution of genotypes, as well as to the genotypes shared among populations (S4A Fig).

A similar pattern was found in the agglomerative hierarchical clustering analysis, which suggests four genetic groups (*K *= 4) with an average silhouette width index *S*_*i*_ = 0.43, indicating a good clustering in the distribution of *A. donax* in Mexico (S4B Fig, S5 Fig). The first cluster (red) consists of 153 individuals from 8 populations belonging to the states of Hidalgo (Huayateno, Venados, San Juan), Michoacán (Tiripetio, Charo, San Lucas Pio), Puebla (Atempan), and San Luis Potosi (Santa Maria del Rio). The second group (blue) includes 92 individuals from three populations (Cococingo, Tres Cruces, and Xilotla) located in the state of Hidalgo and one (Florida) in the state of Michoacán. The third cluster (green), grouped together 172 individuals from populations in the states of Jalisco (Puente, Cocula), Coahuila (Valle Cruya), Veracruz (Escondida, Mancha) and Nayarit (Rio, Montaña). The last group (violet) consists only of 32 individuals belonging to the population of Cuatrocienegas from the state of Coahuila (S4B Fig, S5 Fig). This clustering was also reinforced with a high value of cophenetic correlation (r = 0.88), indicating that the cluster tree reflects the original distances accurately. In this sense, the first two axes of PCA that explained 85% of the total variation support the consistency of four genetic clusters (S5 Fig).

The hierarchical AMOVA analysis based on the four genetic groups described above (Table 3), revealed that most of the genetic variation (73.86%) was partitioned among groups (*F*_*CT*_ = 0.738, P < 0.0001), 14.49% was attributed to differences within populations (Table 3), and the lowest differentiation was found among populations within groups (11.65%). The *F*_*ST*_ was 0.855, which was significantly different from zero at P < 0.0001, thereby indicating high genetic structuring, with more genetic variation partitioned among genetic groups than within populations (Table 3). The coancestry coefficient *θ* = 0.830 (95% C.I = 0.729–0.910) also showed a high genetic structure among the 20 analyzed populations and was similar to *F*_*ST*_ index obtained from AMOVA.

**Table 3:** Analysis of Molecular Variance (AMOVA) for 20 Populations of *Arundo donax* using ISSRs markers. Four groups of populations were defined according to the hierarchical clustering analysis (groups*1*, *2*, *3*, and *4*, see text). ***P < 0.0001.

Source of variation	Degrees of freedom	Sum of squares	Variance components	Percentage of variation	*F* -statistic
Among groups	3	1189.559	3.68942Va	73.86	*F*_*CT*_ = 0.738***
Among populations within groups	16	215.073	0.58166Vb	11.65	*F*_*SC*_ = 0.445***
Within populations	429	310.521	0.72383Vc	14.49	*F*_*ST*_ = 0.855***
Total	448	1715.154	4.99490		

**Fig 2:**
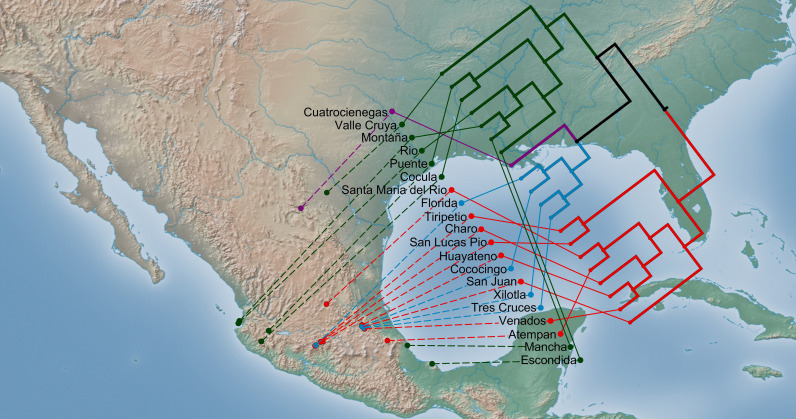
Genetic cluster analysis. Dendrogram of the Unweighted Pair Group Method (UPGMA) cluster analysis based on the Euclidean distances, projected to a geographical environment illustrating genetic and geographical relationships among the 20 populations of *Arundo donax*, with cophenetic correlation = 0.912. Colors indicate the genetic group: red = cluster 1, blue = cluster 2, green = cluster 3, and violet = cluster 4. Made with Natural Earth. Free vector and raster map data @ naturalearthdata.com.

The UPGMA dendrogram based on Euclidean distances at the population level (Fig 2) showed a similar pattern as described above using all the individuals. The largest group (red in Fig 2) includes 8 populations from central Mexico and corresponds to group *1* in S4B Fig. The populations Cococingo, Tres Cruces, Xilotla, and Florida (in blue in S4 Fig), were more similar among them, forming a clade that corresponds to cluster *2* found in S4B Fig. Populations from the states of Jalisco, Coahuila (Valle Cruya), Veracruz and Nayarit form a distinct unit in the dendrogram (green in Fig 2), that is equivalent to the cluster *3* in S4B Fig. All samples from the population Cuatrocienegas remained separated forming a group in violet in Fig 2, which is the same as cluster *4* previously mentioned (S4B Fig).

### Ecological and genotypic characterizations

The ordination analysis of the climatic variables (PCA) indicates that the first principal component summarizes 53.3% of the total variance. The environmental variables most closely correlated to this component were Minimum Temperature of the Coldest Month (bio_6), Precipitation of the Warmest Quarter (bio_18), Mean Temperature of the Driest Quarter (bio_9), Precipitation of the Wettest Quarter (bio_16), Precipitation of the Wettest Month (bio_13), and Mean Temperature of the Coldest Quarter (bio_11). The Mean Diurnal Range (bio_2) and Altitude were found with a negative correlation (S6 Fig). The second principal component summarizes 23.9% of the total climatic variance. The environmental variables with higher correlation with this second component were Temperature Seasonality (bio_4), Maximum Temperature of the Warmest Month (bio_5), Mean Temperature of the Warmest Quarter (bio_10), Mean Temperature of the Wettest Quarter (bio_8), and Isothermality (bio_3), with a negative correlation (S6 Fig). The third component is relatively important, since it summarizes 17.2% of the total variance; the variables correlated to this component were the Precipitation Seasonality (bio_15), and negatively correlated were the Precipitation of the Driest Month (bio_14) and Precipitation of the Driest Quarter (bio_17).

The first principal component separates populations in Central Highlands of Mexico from Coastal populations (S6 Fig). The altitude in central populations (Huayateno, Venados, San Juan, Cococingo, Tres Cruces, Xilotla, Santa Maria Del Rio, Tiripetio, Charo, San Lucas Pio, and Florida) is generally high, with temperatures that vary throughout the year, e.g., they are relatively low in dry (March, and April) and cold (December, January and February) seasons, and temperate during the warm (April, May and June) and wet (July, August and September) months, with low rainfall in the cold season, but moderate in the warm and wet seasons (S6 Fig). All of these populations are grouped into the genetic clusters *1* and *2* (see, S4 Fig and Fig 2). Populations on both coasts of Mexico, Pacific coast (Rio, Montaña, Puente, and Cocula) and Gulf of Mexico coast (Escondida, and Mancha), have moderate temperatures in dry, warm, and wet seasons, with a slight decrease in cold season, and rainfall ranging from high in warm and wet months, to low in cold season. The exception was Escondida population, where the precipitation is high throughout the year, particularly in wet and cold seasons (S6 Fig). These populations are included within the genetic group *3* (see, S4 Fig and Fig 2).

The second principal component mainly distinguishes populations in the North region (Valle Cruya, belonging to the genetic cluster *3*, and Cuatrocienegas, which forms the genetic group *4*). These populations are characterized by a strong variation in temperature at different seasons (i.e., high temperatures in warm and wet seasons, and low temperatures in dry and cold seasons), average altitudes and scarce rainfall (S6 Fig). Finally, the population Atempan from the state of Puebla, from genetic cluster *1* has relatively high altitude with low temperature variability (isothermality), that is, temperatures generally are low throughout the year, with moderate seasonal precipitations (S6 Fig).

To identify the major environmental variables related to genotype distribution, we performed a canonical correspondence analysis (CCA). Among the 20 environmental variables included in the analysis, three had a significant (*P* < 0.05) influence on the genotype data, as suggested by the forward selection option: Temperature Seasonality (bio_4), Mean Temperature of Driest Quarter (bio_9), and Altitude (S7A and S7B Fig). The first two axes explained 70% of the total variance in the data, with 35.5% for axis 1 and 34.5% for axis 2. In all cases the test of significance (Monte Carlo permutation test) was significant for the individual constrained axis, and variation was explained by individual environmental variables (ANOVA P < 0.001).

From the CCA analysis, we can see that populations in the states of Hidalgo, Michoacán, San Luis Potosi, and Puebla were influenced by the altitude (S7A Fig). At the same time, we found that the genotypes distributed in these states were also highly correlated with altitude (S7B Fig) and clearly separates this Central Highlands region from the rest (North and Coasts) regions. Thus, genotypes which are distributed in the Mexican Central Highlands could be mainly characterized by high altitudes and low temperatures (S7 Fig). Populations belonging to the Coastal regions (top in S7A Fig) were grouped together along the genotypes that are distributed in these regions (top in S7B Fig). Results indicate that the genotypes and populations in the Gulf coast and the Pacific coast are highly correlated with the temperature of the driest quarter, and are not found in higher places, suggesting that these genotypes prefer high temperatures and lower altitudes (S7 Fig). Finally, bio_4 (Temperature Seasonality) appeared as the strongest environmental variable correlated to the North region (S7A Fig) and it is directly associated with genotypes distributed in Valle Cruya and Cuatrocienegas (S7B Fig), suggesting that a strong variation in temperature is the main factor that influences genotype abundance in the north of Mexico (S7 Fig). This pattern was also reflected in the agglomerative hierarchical clustering analysis using the same environmental variables (S8 Fig). This ecological characterization suggests three groups (with an average silhouette width index *S*_*i*_ = 0.67) indicating a good clustering in the distribution of *A. donax* in Mexico (S8 Fig). This clustering was also reinforced with a high value of cophenetic correlation (*r* = 0.85), indicating that the cluster tree reflects the original distances accurately. In this sense, the first two axes of PCA that explained 96.6% of the total variance support the consistency of three clusters (S8 Fig).

**Fig 3:**
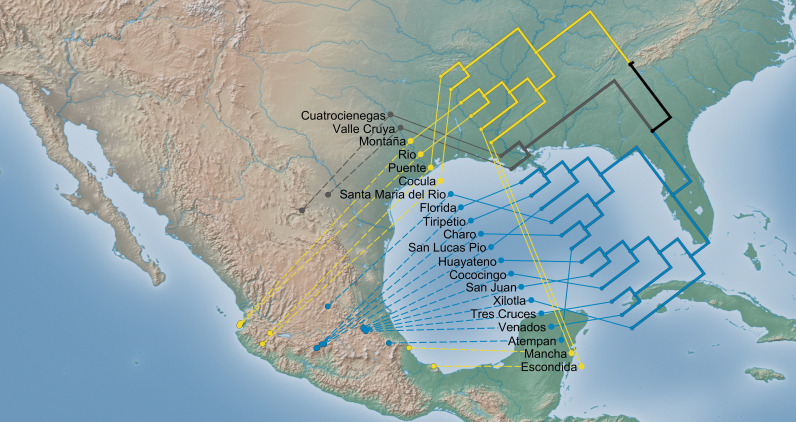
Ecological cluster analysis. Dendrogram of hierarchical clustering analysis depicting the ecological distance among populations and projected to a geographical environment illustrating ecological and geographical relationships among the 20 populations of *Arundo donax*, with cophenetic correlation = 0.85. Colors indicate the optimal number of clusters: blue = Central Highlands, yellow = Coasts, and gray = North. Made with Natural Earth. Free vector and raster map data @ naturalearthdata.com.

All these analyses indicate that the distribution and abundance of genotypes is structured and is influenced by environmental factors and support the fact that in Mexico we have three ecological-genetic groups in *A. donax* (S8 Fig). The first cluster (Central Highlands) consists of 12 populations belonging to the states of Hidalgo, Michoacán, Puebla, and San Luis Potosi (Fig 3). The second cluster (Coasts) comprises populations from the state of Jalisco (Puente, Cocula), Nayarit (Rio, Montaña), and Veracruz (Escondida, Mancha). The third cluster (North) only includes the samples from Cuatrocienegas and Valle Cruya, both from the state of Coahuila (Fig 3).

## Discussion

Here we present the first analysis of genetic diversity in Mexican populations of *A. donax*, along with climatic studies for the species. We detected genetic differentiation and low levels of gene flow among genetic clusters, as well as evidence suggesting that the genetic groups may be adapted to different environmental conditions. Our ecological analyses indicated that in Mexico we have three ecological-genetic groups of *A. donax* populations. Our results add information on the genetic diversity of the species and their populations, and shed light on the complexity of its climatic niche dynamics.

### Clonality and comparisons with other studies of the species

ISSRs have been successfully used as an effective tool to estimate the extent of genetic diversity at inter and intra-specific levels in a wide range of plants, including several studies with invasive plants [23,37,68–78]. In the present study, the ISSRs approach provided an effective and robust mean for examining biogeographic relationships and genetic similarities within and among populations of *A. donax* in Mexico. In our sample of 449 individuals, we found 77 different genotypes and a G/N of 0.17. The extent of polymorphism found in the ISSRs can be attributed to several factors, for example, according to Pradeep et al. [79 and references therein] **“**mutations at the priming site (i.e., the microsatellite) could prevent amplification of a fragment and thus give a presence or absence of bands (polymorphism). Usually, di-nucleotide repeats of primers anchored either at 3’ or 5’ end (like the ones used here) reveal high polymorphism, also, an insertion or deletion event within the microsatellite region or the amplified region would result in the absence of a product or length polymorphism**”**. In general, primers with (CT), (TC), (AG), or such as those we use here (GA), (AC), and (CA) repeats show higher polymorphism than primers with other di-, tri- or tetra-nucleotide repeats [79 and references therein].

In Table 1 we included the values of the proportion of distinguishable genotypes (G/N) obtained in other studies in *A. donax* in various parts of the world using different genetic markers. These results include different estimates of the levels of diversity around the world, ranging from a single genetic clone in the United States, southern France [30], Italy [23,36], the Mediterranean basin [38], South Africa [34], and Australia [28], to being moderately diverse in another study in the United States [29] and Mexico (this study), and reaching a high level of genotypic variation in a report from southeastern Australia [37].

All this evidence indicates that the history of *A. donax* is complicated, and suggests different evolutionary and invasion dynamics when we compare our data with data from the United States [30], Europe, and mainly with the Mediterranean region [23,38,36], and likely suggests different origins of the Mexican and United States *A. donax* populations.

The advances in the study of this species indicate that *A. donax* is native to Asia [23–25], with subsequent dispersion throughout the Middle East to southern Europe and Africa. From the Mediterranean region *A. donax* was apparently introduced into the United States for multiple uses in the 19th century [19,23]. This pattern of dispersion produced nested founder events, which combined with the selection of specific variants with desirable traits for cultivation and propagation (length of culms, sterility, etc.), may have resulted in a substantial reduction of its genetic diversity during its spread [23,30]. In addition, the apparently obligate asexual reproduction in the introduced ranges of the Mediterranean region, Europe and United States, would also contribute in keeping a low genetic diversity [30].

Unlike the previously described patterns for the USA populations, our results indicate that all the analyzed Mexican populations were multiclonal (each population including from 3 to 9 different genotypes), and that their genetic diversity is high compared to some populations in other parts of the world (Table 1), which is in part maintained by different modes of dispersal (i.e., clonal propagation by means of broken stems or rhizome fragments vs. some levels of sexual reproduction), as well as it suggests sporadic sexual reproductive events are likely to happen, and perhaps, are variable among the Mexican *A. donax* populations (Table 1).

It is important to note that our study is the one with the largest sample size, allowing us to detect more genotypes. This large sample size may have led us to infer that the Mexican populations are less clonal and more variable than many other populations (Table 1). But, since the different studies were made with different sampling designs and molecular markers, it is difficult to conclude more at this moment, if indeed *A. donax* is mainly clonal, although in many studies several genotypes were detected.

We consider that it will be important to use standardized methods to study the species worldwide, maybe using a genotyping method related to massive sequencing, that may allow us to replicate the methods and obtain a large number of markers (i.e., GBS, RAD-seq, etc.). Also, it would be important in the future to include large sample sizes from more areas in Asia, where the species probably originated [23].

An alternative to sexual reproduction that can explain the level of variation observed in *A. donax* could be multiple introductions from different source regions, as was suggested by Khudamrongsawat et al. [29], and evidenced by Tarin et al. [31] in the United States. In addition, somatic mutations may also contribute to some of the genetic variation in *A. donax* [29], but for instance, it would be necessary to compare the genetic composition of plants with known rhizome connections to determine this [29,37].

### Geographical distribution of genotypes and genotypic richness

We found a high regional structure of *A. donax* in Mexico, with a particularly high genotypic richness in the states of Hidalgo and Michoacán. We detected a high frequency of shared genotypes among populations of the Central Highlands region, as well as among populations belonging to both coasts (Pacific and Gulf of Mexico), but not between populations of the different regions (S1 Table).

Additionally, we identified a high frequency of genotypes with a restricted distribution, in particular in the populations from the state of Hidalgo (Cococingo, Tres Cruces, and Xilotla), as well as in the population of Florida, in the state of Michoacán, which does not share genotypes with any of the other populations (S1 Table), and in the two populations of the Northern region (Valle Cruya and Cuatrocienegas) in the state of Coahuila (S1 Table).

### Sexual and asexual reproduction

In plants, angiosperms display a wide range of strategies for achieving both sexual and asexual propagation, and studies have shown that reproductive mode commonly varies among species and among populations within a species [47,80]. Even though *A. donax* produces flowers, viable seeds have not been observed in most areas where it has been introduced [19], including North America [21,81], Europe [26] and Australia [82]. The infertility of the seeds could be due to post-meiotic alterations during the development of the ovules and pollen [23], therefore, vegetative propagation through stem layering and rhizome proliferation [83] is believed to be the primary mode of reproduction of *A. donax* in North America [21,84], Europe [23,26] and southern Australia [37,82].

Here, we evaluated the hypothesis of random mating in *A. donax* by analyzing linkage disequilibrium among loci. We found several linked loci, but also evidence suggesting that there are loci that fall inside of the distribution expected under no linkage (S3 Fig). That is, our results indicate that while asexual propagation is the predominant mating system in half of the analyzed populations (Table 2) -- which is in accordance with the findings from other studies [23,29,30,37]-- there is some sexual reproduction, mainly in populations belonging to the Coasts group, and in some populations (Cococingo, Tres Cruces, Xilotla, and Tiripetio) in the Central Highlands group, as well as in Valle Cruya in the northern region (Table 2). In addition to this, we found that these recombinant populations possess either less or a similar degree of genetic variation to those with predominantly asexual reproduction (Table 2), contrary to the general expectation that sexual reproduction tends to increase population genetic variation [85], suggesting that the reproductive system is not a determining factor in the levels of genetic variation during the range expansion of *A. donax* in Mexico.

Although the frequency of sexual reproductive events in *A. donax* remains unclear, viable seeds have been reported in Asian populations (Afghanistan, southwestern Pakistan, and Iran) [19]. Johnson et al. [84] found a low frequency of ovules that may be viable in florets collected from California, Nevada, Colorado, New Mexico, Texas, Georgia, Washington D. C., and Nuevo Leon (northern Mexico). So, our results of some recombination and in some cases high G/N values are not surprising, but their interpretation should be cautious, since nothing is known about the biological reproduction in the Mexican *A.donax* populations. Eventually, it will be important to conduct field studies on fruit production, seed set rates, viability and germination of seeds, as well as on their ability to spread in different populations in this country.

### Structure and genetic relationship

The distribution and number of genetic variants within and among populations in a new range are strongly affected by the number of introductions, the diversity of the founders, the mating system, life-history traits, and the post-introduction processes of micro-evolutionary forces, such as gene flow, genetic drift and selection [5,12,86,87]. These mechanisms would generate new combinations of genetic variation that are not found in the native range [5,86,88,89]. Analyzing the genetic diversity and structure of introduced populations is a key component to understanding the potential of introduced species to become established and spread in the novel range. It is critical to understand how introductions affect the amount and structure of genetic variation, as well as how variation is partitioned within and between populations (12, 14, 87].

In the case of *A. donax* in Mexico, the ordination analysis (PCo) complemented with agglomerative hierarchical clustering analysis provided a spatial representation of relative similarities and differences among individuals (S4 Fig, S5 Fig). The four clades detected (Fig 2) support the idea of multiple independent introductions of *A. donax* to Mexico, and suggests that the genetic groups may be adapted to different environmental conditions. These genetic groups were also studied with an AMOVA that showed that the highest percentage of variation is found among the four geographic regions, with significant differences among populations within regions (Table 3). This result is consistent with the other analyses (principal coordinate analysis and agglomerative hierarchical clustering analysis) and point out a clear genetic differentiation and low levels of gene flow among clusters, in agreement with the *θ* estimator and *F*_*ST*_ index obtained from AMOVA.

Among multiple-population clustering, genetic group *3* spanned multiple populations that are separated by long distances, whereas the other groups (*1*, *2*, and *4*) were restricted to smaller areas (Fig 2). These results allowed us to define genetic structure in the species in Mexico. Similar results were found in *A. donax* in populations growing in Greece, Italy and southern France, as evidenced by RAPDs, indicating a clustering of the populations in relation to their geographical origin, reflecting a restricted gene flow among geographic regions [26]. Furthermore, based on haplotype diversity, Hardion et al. [24] identified four biogeographic clusters distributed from the Mediterranean basin to the Eastern Himalaya-China region. On the other hand, in a more recent study using nuclear and plastid loci, Zecca et al. [25] found the presence of six lineages in *A. donax* distributed from the Asian regions to the Mediterranean basin. A possible reason for the strong genetic structure of *A. donax* in Mexico is that populations may have not reached the gene flow–genetic drift equilibrium, because of their relatively recent introduction from other populations, but we do not have information about this at the present.

### Ecological and genotypic characterizations

A PCA allowed the identification of climatic space conditions that determine the distribution of populations of giant reed and permitted a comparison of the ecological climatic preferences among all genetic groups (S6 Fig). Multivariate statistical techniques, such as CCA analysis, coupled with genetic data, allowed us to evaluate the possible role of environmental conditions between populations and to identify the major environmental variables (Temperature Seasonality, Mean Temperature of the Driest Quarter, and Altitude) that determine the distribution and abundance of the 77 genotypes of *Arundo* in Mexico (S7 Fig).

Because genetic variation provides the raw materials for adaptive evolution, it is critical to understand how introductions affect the amount and structure of genetic variation. The agglomerative hierarchical clustering analysis (S8 Fig) carried out using the three previously selected environmental variables that explain the current distribution of genotypes, support the hypothesis that in Mexico we have three main ecological-genetic groups (Central Highlands, Coasts and North), which each may represent different independent introduction events (Fig 3). In addition, our data indicate that most genotypes (59.74%) were restricted to the Central Highlands, followed by Coast and North region, with 23.38% and 16.88% respectively.

All the above suggests that the genotypes found in the Mexican Central Highlands are adapted to higher altitudes and lower temperatures, while genotypes from the Coast regions are adapted to higher temperatures and medium to low elevations (S7 Fig and Fig 3). On the other hand, the genotypes distributed in the North region are adapted to stronger seasonality with scarce rainfall (S7 Fig and Fig 3).

However, the observed pattern of high regional differentiation may be the outcome of the random establishment of genotypes in different areas mediated by human dispersal, since this species is very commonly found along roads, on the shores of lakes, rivers and ponds, or even in swamps and wetlands usually associated with human disturbance. Reciprocal common garden experiments will be needed to better understand the degree of local adaptation and niche boundaries of genotypes of *A. donax*.

## Concluding remarks and perspectives

This study represents the first detailed analysis on the ecological characterization of genotypes of *Arundo* in Mexico and provides an initial estimate of its genetic variation. We were able to identify that the distribution and abundance of the different genotypes in the populations analyzed are influenced by fluctuations in temperature and altitude ranges.

In particular, our analyses suggest that multiple introductions of *A. donax* may have occurred in Mexico, and maybe these introduced genotypes came from different disjunct regions. The results are consistent with two or more introductions. However, given the lack of analyzed samples from other regions in our study (i.e., from United States, Europe, and Asia), we were unable to pinpoint the origins of those introductions. Future comparisons of the genetic diversity of *A. donax* in the introduced and the native range of populations using the same genetic marker and similar samplings will provide valuable information about the historical process of introduction, including founder events, genetic bottlenecks and selection [5,12,90], and will also help us to explain the observed patterns in the genetic and ecological structure obtained here.

In recent years, efforts have been made to identify specific phytophagous insects as biocontrol agents in United States [91–94]. However, the high regional differentiation of giant reed in Mexico and the multiple genotypes detected implies that different populations may have different levels of susceptibility or resistance to pathogens or other biocontrol agents. Classical biological control using natural enemies from its native range may prove to be more complicated than previously thought. For instance, if populations from Mexico and United Stated are highly divergent and they come from multiple different locations, biocontrol may not be a practical tool for the management of Mexican lineages of *A. donax*, unless biocontrol agents are also brought from these multiple locations. Also, it must be considered before promoting its removal in Mexico, that *A. donax* is still an important plant with many uses in rural communities of Mexico, and in some cases may be even actively propagated by the people (Pers. Obs.).

In addition, further research of biogeographic relationships in *A. donax* should include populations across a broader range from North America (USA and Mexico), Europe (mainly from Mediterranean basin), and Asia (e.g., Afghanistan, Iran, Pakistan, China). In these future studies it will be important to also use different molecular tools, for instance, chloroplast DNA [24] could also be used in conjunction with ISSRs or microsatellites [31] or SNPs data derived from massive sequencing analysis. It will be important to analyze these data coupled with detailed ecological analysis (for instance multivariate statistical analyses and ecological niche modeling), to establish more specific hypotheses that eventually will allow us to investigate and to identify if there are different lineages throughout the global distribution and to determine the possible divergences between those lineages.

These future studies will also be relevant to establish the degree in which the climatic niche of the introduced ranges resembles (niche conservatism) or differs (niche shift) from the native range, and evaluate the role of biologically relevant climatic factors that influence the evolutionary history of populations.

In particular, we consider that a detailed genomic study would be important to identify candidate loci under selection and to advance in analyses of local adaptation, coupled with field experiments (i.e., transplants among populations with different climatic conditions). Such information is crucial for a thorough understanding of evolutionary ecology and to distinguish the roles of genetic drift and selection, as well as address in detail the origin of *A. donax* in Mexico. We will also need the ecological and morphological studies to estimate the role and proportion of sexual reproduction in the different populations.

## Supporting information

S1 Table Geographical distribution of 77 genotypes found in the populations analyzed of *Arundo donax* in Mexico. In bold are shown genotypes found in one population (unique genotypes). Populations in italics and bold depict populations that do not shared genotypes. Gen = Genotype.
(PDF)

S1 Fig Geographical distribution of seventy-seven genotypes found in 20 populations of *Arundo donax* in Mexico. Colors indicate different genotypes. Made with Natural Earth. Free vector and raster map data @ naturalearthdata.com.
(PDF)

S2 Fig Genotype accumulation curve for 449 analyzed samples of *Arundo donax.* The horizontal axis represents the number of loci randomly sampled without replacement up to *n *− 1 loci, the vertical axis shows the number of unique genotypes observed in the data set (77). The red dashed line represents 100% of the total observed genotypes and blue line correspond to the trendline.
(PDF)

S3 Fig Visualizations of tests for linkage disequilibrium, the matrix showing the index of association in a pairwise manner among all loci. rBarD is expected to be zero if populations freely recombine and significantly greater than zero if association between alleles is non-random (clonality).
(PDF)

S4 Fig Genetic clustering analysis of ISSRs data in 449 samples of *Arundo donax.* In (A) Principal coordinates analysis (PCoA) based on Euclidean distances. The first two coordinates explain 68.72% (54.27% and 14.45%) of the total variance. In (B) Dendrogram of hierarchical clustering analysis depicting the genetic distance between all samples analyzed, with cophenetic correlation = 0.88. Colors indicate the optimal number of genetic clusters: red = cluster 1, blue = cluster 2, green = cluster 3, and violet = cluster 4.
(PDF)

S5 Fig Agglomerative hierarchical clustering analysis. In (A) the optimal number of clusters determined by means of average silhouette method. In (B) the assessing the goodness of clustering, with average silhouette width *si* = 0.43. In (C) Cluster plot of the ordination analysis (PCA) showing differentiation among genetic clusters, the first two components explain 85% of the total variation in the data. Colors in (B) and (C) indicate the optimal number of clusters.
(PDF)

S6 Fig Biplot of Principal Component Analysis (PCA) depicting the differences in the environmental space among 20 populations analyzed. The first two components explain 77.2% of the total variation in the samples.
(PDF)

S7 Fig Correlation biplot of canonical correspodence analysis (CCA) ordinations of the 20 populations based on genotype distribution data. Only the environmental variables retained by forward selection (*p* < 0.05) are shown. The first two axes explain 70% of the total variation in the data. In (A) Correlation of populations. In (B) Correlation of geographical distribution of genotypes, bio_4 = Temperature Seasonality, bio_9 = Mean Temperature of Driest Quarter. Colors in (A) indicate the genetic group: red = cluster 1, blue = cluster 2, green = cluster 3, and violet = cluster 4.
(PDF)

S8 Fig Ecological characterization performed with the previously selected bioclimatic variables from CCA (bio_4, bio_9, and Altitude). In (A) Dendrogram of hierarchical clustering analysis depicting the ecological distance between populations, with cophenetic correlation = 0.85. In (B) Cluster plot of the ordination analysis (PCA) showing differentiation of the environmental space among ecological clusters, the main two components explain 96.6% of the total variation. In (C) the optimal number of clusters determined by means of average silhouette method. In (D) the assessing the goodness of clustering, with average silhouette width *si* = 0.67. Colors indicate the optimal number of ecological clusters.
(PDF)
