# Supplementary material for: Genetic and ecological characterization of the giant reed (Arundo donax) in Central Mexico
Source: PLoS One. 2025 May 7;20(5):e0319214. doi: 10.1371/journal.pone.0319214 (PMC12057871; doi:10.1371/journal.pone.0319214)
Supplement: S1 Table — In bold are shown genotypes found in one population (unique genotypes). Populations in italics and bold depict populations that do not shared genotypes. Gen = Genotype. (PDF) [file pone.0319214.s001.pdf]

Genetic and ecological characterization of the giant reed (*Arundo donax*) in Central Mexico

Ricardo Colin, Erika Aguirre-Planter and Luis E. Eguiarte

Appendix (Supplemental Data)

S1 Table. Geographical distribution of 77 genotypes found in the populations analyzed of *Arundo donax* in Mexico. In bold are shown genotypes found in one population (unique genotypes). Populations in italics and bold depict populations that do not shared genotypes. Gen = Genotype.

Population	Code	State	Genotypes
1. Huayateno	HUAY	Hidalgo	Gen_1, Gen_2, Gen_3, Gen_4, Gen_5, Gen_6
2. Venados	VENA	Hidalgo	Gen_1, Gen_2, Gen_3, Gen_7, **Gen_8**
3. San Juan	JUAN	Hidalgo	Gen_2, Gen_3, Gen_4, Gen_6, Gen_7, **Gen_9, Gen_10, Gen_11**
4. Cococingo	COC	Hidalgo	**Gen_12, Gen_13, Gen_14, Gen_15**
5. Tres Cruces	CRUC	Hidalgo	**Gen_16, Gen_17, Gen_18, Gen_19**
6. Xilotla	XIL	Hidalgo	**Gen_20, Gen_21, Gen_22**
7. Santa Maria del Rio	SAN	San Luis Potosi	Gen_1, Gen_2, **Gen_23, Gen_24, Gen_25, Gen_26**, Gen_27**, Gen_28, Gen_29**
8. Atempan	ATE	Puebla	Gen_1, Gen_2, Gen_3, Gen_7, Gen_27, **Gen_30, Gen_31**
9. Tiripetio	TIR	Michoacán	Gen_27, Gen_32, Gen_33, **Gen_34, Gen_35**
10. Charo	CHA	Michoacán	Gen_1, Gen_2, Gen_5, Gen_7, Gen_27, Gen_32, **Gen_36, Gen_37**
11. San Lucas Pio	LUC	Michoacán	Gen_27, Gen_33, **Gen_38, Gen_39, Gen_40**
12. Florida	FLO	Michoacán	**Gen_41, Gen_42, Gen_43, Gen_44, Gen_45, Gen_46**
13. Puente	PUE	Jalisco	**Gen_47**, Gen_48, Gen_49, Gen_50, Gen_51, Gen_52, Gen_53, Gen_54, Gen_55
14. Cocula	COA	Jalisco	Gen_48, Gen_49, Gen_50, Gen_51, Gen_52, Gen_53, Gen_54, Gen_55, Gen_56, **Gen_57**
15. Valle Cruya	VAL	Coahuila	**Gen_58, Gen_59, Gen_60, Gen_61, Gen_62, Gen_63, Gen_64, Gen_65**
			**Gen_66, Gen_67, Gen_68, Gen_69, Gen_70**

16. Cuatrocienegas	CCC	Coahuila	
17. Escondida	ESC	Veracruz	Gen_53, Gen_56, Gen_71, Gen_72, Gen_73, **Gen_74**
18. Mancha	MAN	Veracruz	Gen_53, Gen_56, Gen_71, Gen_72, Gen_73, Gen_75
19. Rio	RIO	Nayarit	Gen_53, Gen_56, Gen_71, Gen_73, Gen_75, **Gen_76, Gen_77**
20. Montaña	MON	Nayarit	Gen_56, Gen_72, Gen_75
