# Supplementary material for: Genetic and ecological characterization of the giant reed (Arundo donax) in Central Mexico
Source: PLoS One. 2025 May 7;20(5):e0319214. doi: 10.1371/journal.pone.0319214 (PMC12057871; doi:10.1371/journal.pone.0319214)
Supplement: S1 Fig — Colors indicate different genotypes. Made with Natural Earth. Free vector and raster map data @ naturalearthdata.com. (PDF) [file pone.0319214.s002.pdf]

# Genetic and ecological characterization of the giant reed (*Arundo donax*) in Central Mexico

Ricardo Colin, Erika Aguirre-Planter and Luis E. Eguiarte

## Appendix (Supplemental Data)

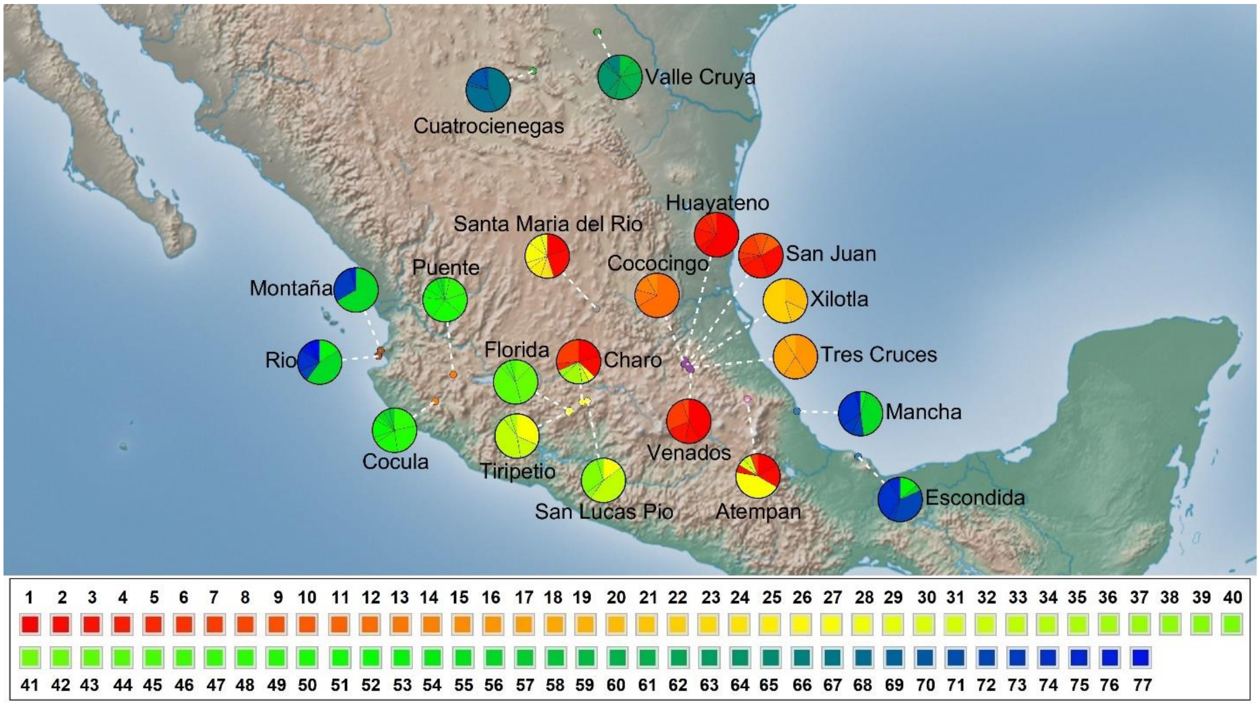


**S1 Fig. Geographical distribution of seventy-seven genotypes found in 20 populations of *Arundo donax* in Mexico**. Colors indicate different genotypes. Made with Natural Earth. Free vector and raster map data @ naturalearthdata.com.
