# Supplementary material for: Genetic and ecological characterization of the giant reed (Arundo donax) in Central Mexico
Source: PLoS One. 2025 May 7;20(5):e0319214. doi: 10.1371/journal.pone.0319214 (PMC12057871; doi:10.1371/journal.pone.0319214)
Supplement: S2 Fig — The horizontal axis represents the number of loci randomly sampled without replacement up to n − 1 loci, the vertical axis shows the number of unique genotypes observed in the data set (77). The red dashed line represents 100% of the total observed genotypes and blue line correspond to the trendline. (PDF) [file pone.0319214.s003.pdf]

Genetic and ecological characterization of the giant reed (*Arundo donax*) in Central Mexico

Ricardo Colin, Erika Aguirre-Planter and Luis E. Eguiarte

Appendix (Supplemental Data)

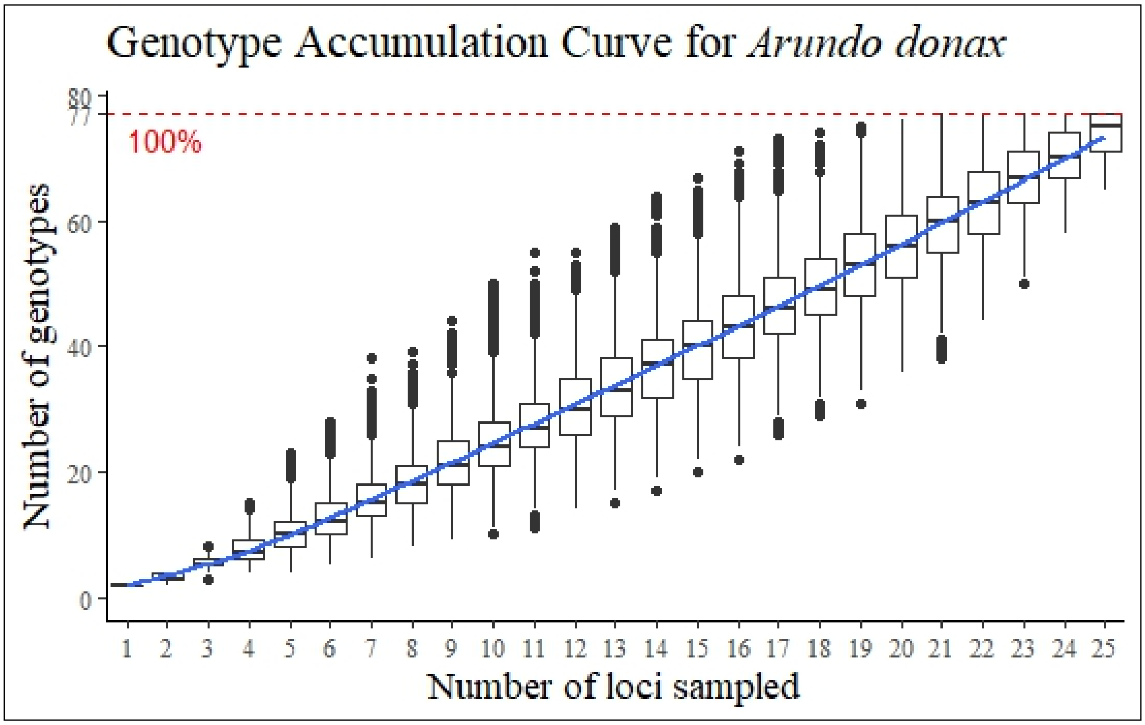

S2 Fig. Genotype accumulation curve for 449 analyzed samples of *Arundo donax*. The horizontal axis represents the number of loci randomly sampled without replacement up to $n - 1$ loci, the vertical axis shows the number of unique genotypes observed in the data set (77). The red dashed line represents 100% of the total observed genotypes and blue line correspond to the trendline.
