# Supplementary material for: Genetic and ecological characterization of the giant reed (Arundo donax) in Central Mexico
Source: PLoS One. 2025 May 7;20(5):e0319214. doi: 10.1371/journal.pone.0319214 (PMC12057871; doi:10.1371/journal.pone.0319214)
Supplement: S3 Fig — rBarD is expected to be zero if populations freely recombine and significantly greater than zero if association between alleles is non-random (clonality). (PDF) [file pone.0319214.s004.pdf]

# Genetic and ecological characterization of the giant reed (*Arundo donax*) in Central Mexico

Ricardo Colin, Erika Aguirre-Planter and Luis E. Eguiarte

## Appendix (Supplemental Data)

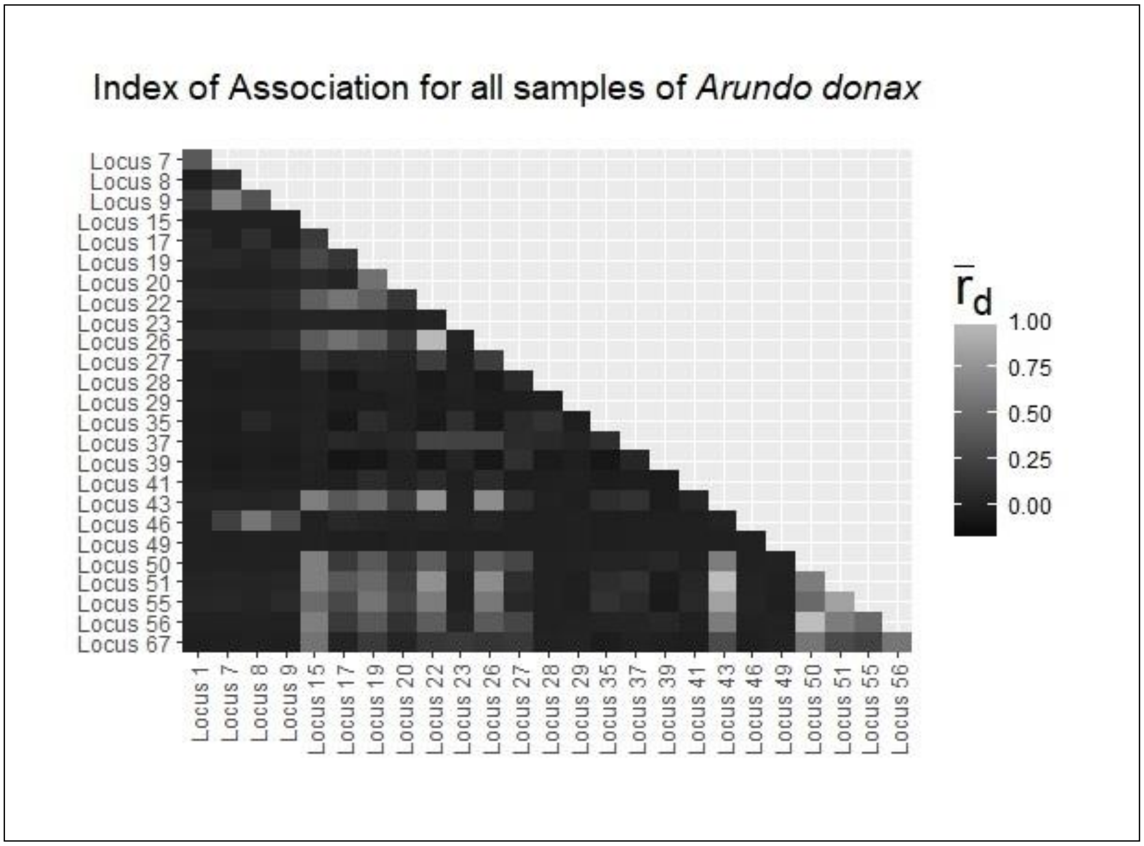


**S3 Fig. Visualizations of tests for linkage disequilibrium, the matrix showing the index of association in a pairwise manner among all loci.** rBarD is expected to be zero if populations freely recombine and significantly greater than zero if association between alleles is non-random (clonality).
