# Supplementary material for: Genetic and ecological characterization of the giant reed (Arundo donax) in Central Mexico
Source: PLoS One. 2025 May 7;20(5):e0319214. doi: 10.1371/journal.pone.0319214 (PMC12057871; doi:10.1371/journal.pone.0319214)
Supplement: S4 Fig — In (A) Principal coordinates analysis (PCoA) based on Euclidean distances. The first two coordinates explain 68.72% (54.27% and 14.45%) of the total variance. In (B) Dendrogram of hierarchical clustering analysis depicting the genetic distance between all samples analyzed, with cophenetic correlation = 0.88. Colors indicate the optimal number of genetic clusters: red = cluster 1, blue = cluster 2, green = cluster 3, and violet = cluster 4. (PDF) [file pone.0319214.s005.pdf]

Genetic and ecological characterization of the giant reed (*Arundo donax*) in Central Mexico

Ricardo Colin, Erika Aguirre-Planter and Luis E. Eguiarte

Appendix (Supplemental Data)

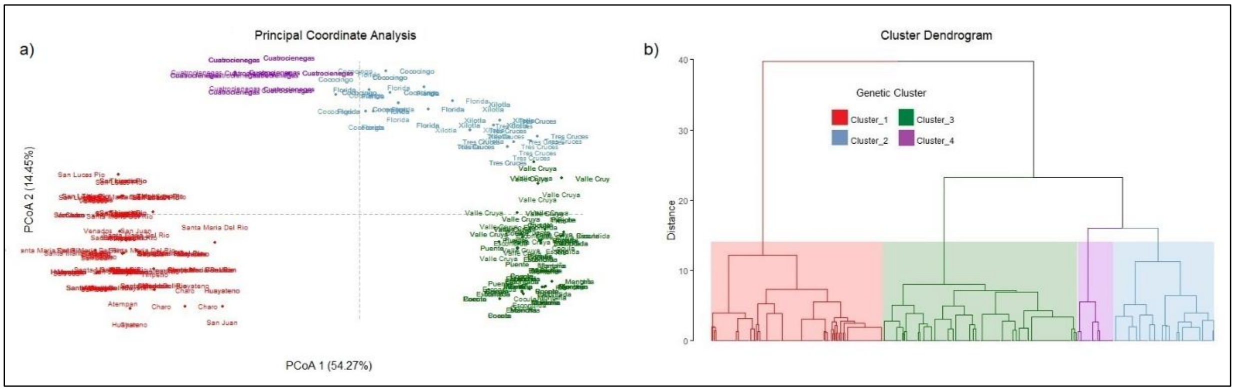

S4 Fig. Genetic clustering analysis of ISSRs data in 449 samples of *Arundo donax*. In (A) Principal coordinates analysis (PCoA) based on Euclidean distances. The first two coordinates explain 68.72% (54.27% and 14.45%) of the total variance. In (B) Dendrogram of hierarchical clustering analysis depicting the genetic distance between all samples analyzed, with cophenetic correlation = 0.88. Colors indicate the optimal number of genetic clusters: red = cluster 1, blue = cluster 2, green = cluster 3, and violet = cluster 4.
