# Supplementary material for: Genetic and ecological characterization of the giant reed (Arundo donax) in Central Mexico
Source: PLoS One. 2025 May 7;20(5):e0319214. doi: 10.1371/journal.pone.0319214 (PMC12057871; doi:10.1371/journal.pone.0319214)
Supplement: S5 Fig — In (A) the optimal number of clusters determined by means of average silhouette method. In (B) the assessing the goodness of clustering, with average silhouette width si = 0.43. In (C) Cluster plot of the ordination analysis (PCA) showing differentiation among genetic clusters, the first two components explain 85% of the total variation in the data. Colors in (B) and (C) indicate the optimal number of clusters. (PDF) [file pone.0319214.s006.pdf]

Genetic and ecological characterization of the giant reed (*Arundo donax*) in Central Mexico

Ricardo Colin, Erika Aguirre-Planter and Luis E. Eguiarte

Appendix (Supplemental Data)

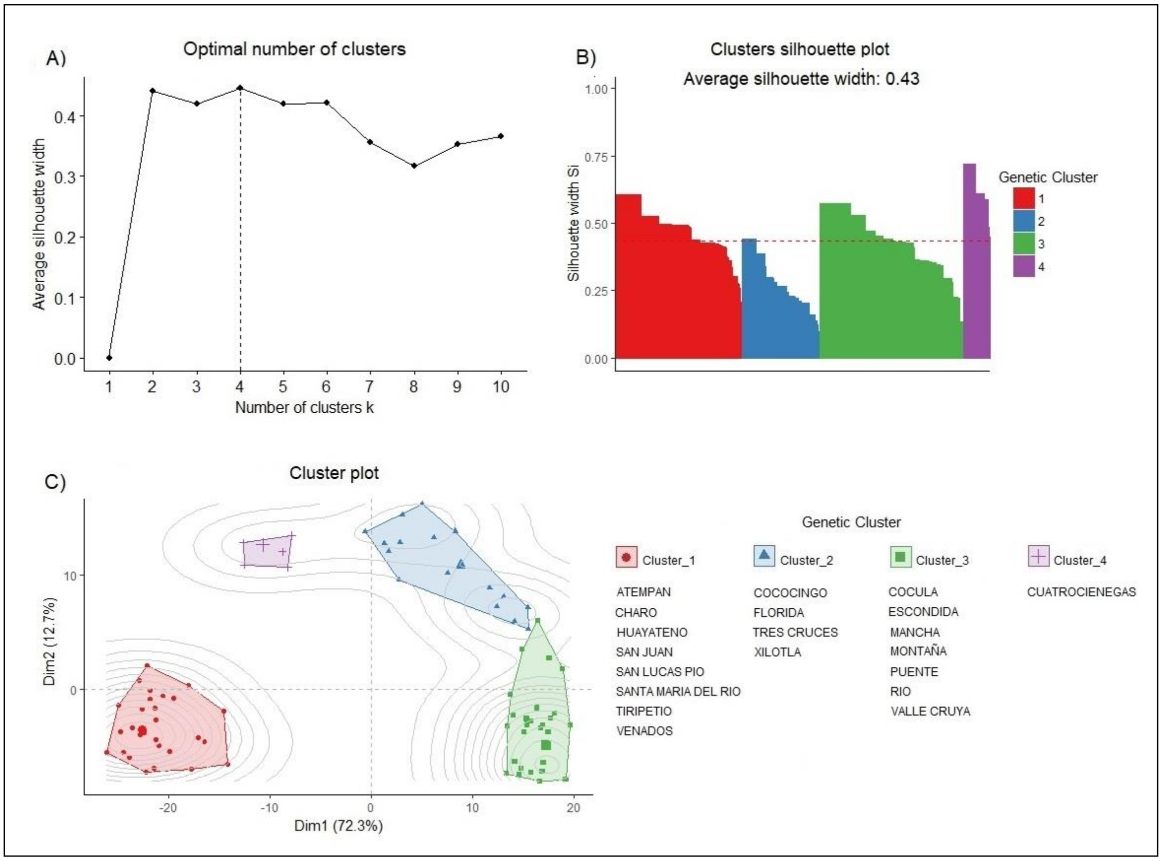

S5 Fig. Agglomerative hierarchical clustering analysis. In (A) the optimal number of clusters determined by means of average silhouette method. In (B) the assessing the goodness of clustering, with average silhouette width *si* = 0.43. In (C) Cluster plot of the ordination analysis (PCA) showing differentiation among genetic clusters, the first two components explain 85% of the total variation in the data. Colors in (B) and (C) indicate the optimal number of clusters.
