# Supplementary material for: Genetic and ecological characterization of the giant reed (Arundo donax) in Central Mexico
Source: PLoS One. 2025 May 7;20(5):e0319214. doi: 10.1371/journal.pone.0319214 (PMC12057871; doi:10.1371/journal.pone.0319214)
Supplement: S6 Fig — The first two components explain 77.2% of the total variation in the samples. (PDF) [file pone.0319214.s007.pdf]

Genetic and ecological characterization of the giant reed (*Arundo donax*) in Central Mexico

Ricardo Colin, Erika Aguirre-Planter and Luis E. Eguiarte

Appendix (Supplemental Data)

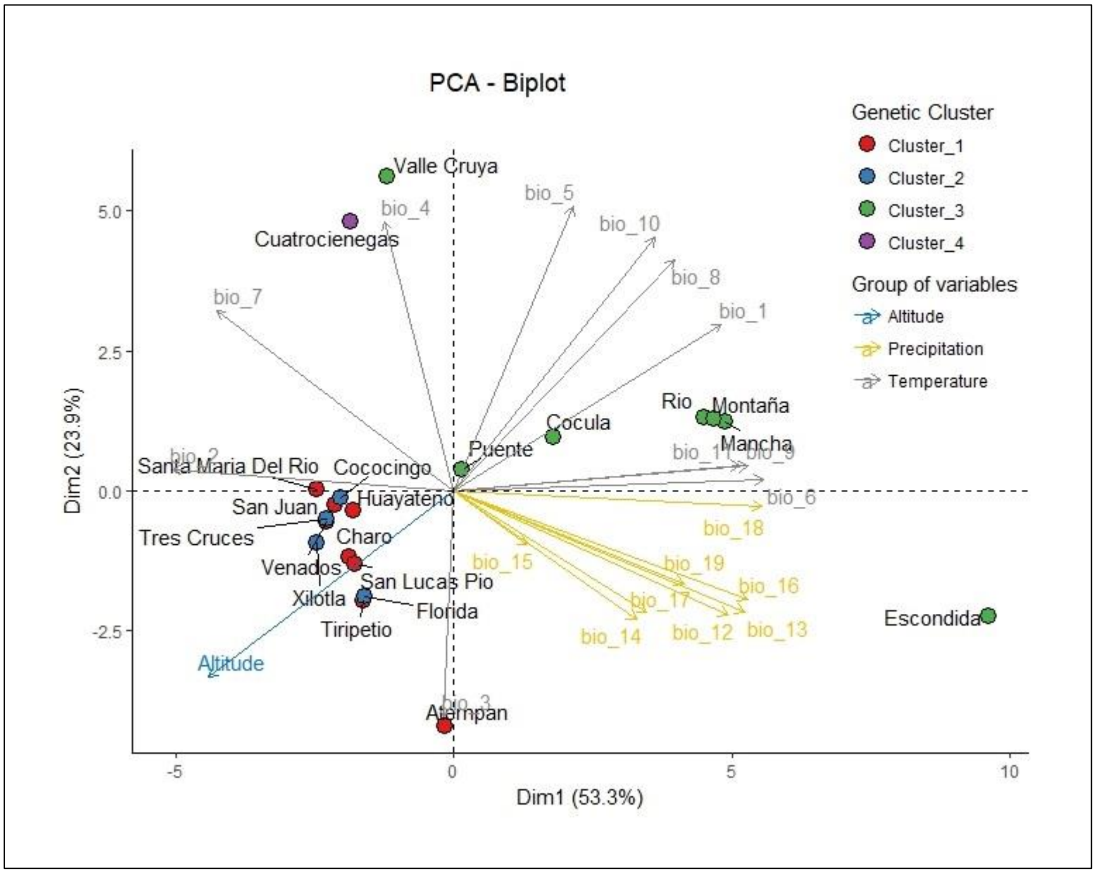

S6 Fig. Biplot of Principal Component Analysis (PCA) depicting the differences in the environmental space among 20 populations analyzed. The first two components explain 77.2% of the total variation in the samples.
