# Supplementary material for: Genetic and ecological characterization of the giant reed (Arundo donax) in Central Mexico
Source: PLoS One. 2025 May 7;20(5):e0319214. doi: 10.1371/journal.pone.0319214 (PMC12057871; doi:10.1371/journal.pone.0319214)
Supplement: S7 Fig — Only the environmental variables retained by forward selection (p < 0.05) are shown. The first two axes explain 70% of the total variation in the data. In (A) Correlation of populations. In (B) Correlation of geographical distribution of genotypes, bio_4 = Temperature Seasonality, bio_9 = Mean Temperature of Driest Quarter. Colors in (A) indicate the genetic group: red = cluster 1, blue = cluster 2, green = cluster 3, and violet = cluster 4. (PDF) [file pone.0319214.s008.pdf]

Genetic and ecological characterization of the giant reed (*Arundo donax*) in Central Mexico

Ricardo Colin, Erika Aguirre-Planter and Luis E. Eguiarte

Appendix (Supplemental Data)

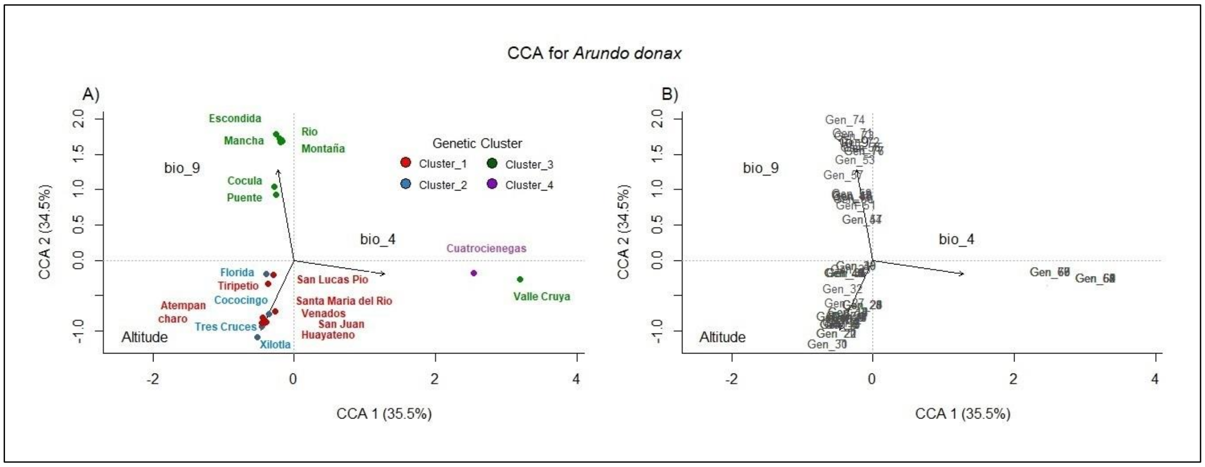

S7 Fig. Correlation biplot of canonical correspodence analysis (CCA) ordinations of the 20 populations based on genotype distribution data. Only the environmental variables retained by forward selection ($p < 0.05$) are shown. The first two axes explain 70% of the total variation in the data. In (A) Correlation of populations. In (B) Correlation of geographical distribution of genotypes, bio_4 = Temperature Seasonality, bio_9 = Mean Temperature of Driest Quarter. Colors in (A) indicate the genetic group: red = cluster 1, blue = cluster 2, green = cluster 3, and violet = cluster 4.
