# Supplementary material for: Genetic and ecological characterization of the giant reed (Arundo donax) in Central Mexico
Source: PLoS One. 2025 May 7;20(5):e0319214. doi: 10.1371/journal.pone.0319214 (PMC12057871; doi:10.1371/journal.pone.0319214)
Supplement: S8 Fig — In (A) Dendrogram of hierarchical clustering analysis depicting the ecological distance between populations, with cophenetic correlation = 0.85. In (B) Cluster plot of the ordination analysis (PCA) showing differentiation of the environmental space among ecological clusters, the main two components explain 96.6% of the total variation. In (C) the optimal number of clusters determined by means of average silhouette method. In (D) the assessing the goodness of clustering, with average silhouette width si = 0.67. Colors indicate the optimal number of ecological clusters. (PDF) [file pone.0319214.s009.pdf]

Genetic and ecological characterization of the giant reed (*Arundo donax*) in Central Mexico

Ricardo Colin, Erika Aguirre-Planter and Luis E. Eguiarte

Appendix (Supplemental Data)

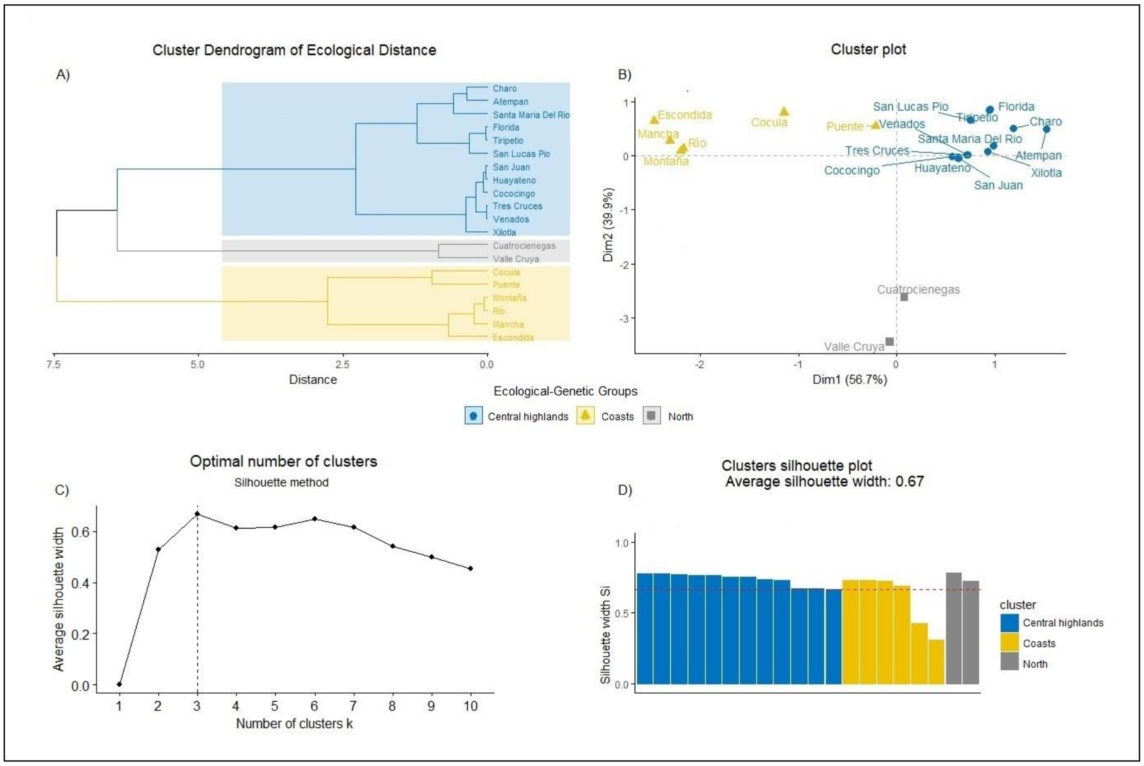

S8 Fig. Ecological characterization performed with the previously selected bioclimatic variables from CCA (bio_4, bio_9, and Altitude). In (A) Dendrogram of hierarchical clustering analysis depicting the ecological distance between populations, with cophenetic correlation = 0.85. In (B) Cluster plot of the ordination analysis (PCA) showing differentiation of the environmental space among ecological clusters, the main tow components explain 96.6% of the total variation. In (C) the optimal number of clusters determined by means of average silhouette method. In (D) the assessing the goodness of clustering, with average silhouette width *si* = 0.67. Colors indicate the optimal number of ecological clusters.
